# Rescue endoscopic vacuum therapy after failed surgical repair of a perforated gastric ulcer

**DOI:** 10.1055/a-2829-5064

**Published:** 2026-07-08

**Authors:** Joana Mota, Joel Ferreira-Silva, Guilherme Macedo, Eduardo Rodrigues-Pinto

**Affiliations:** 1 Gastroenterology Department 285211 Centro Hospitalar Universitário de São JoãoPortoPortugal; 2 Faculty of Medicine 26706 University of PortoPortoPortugal

**Fig. 1:**
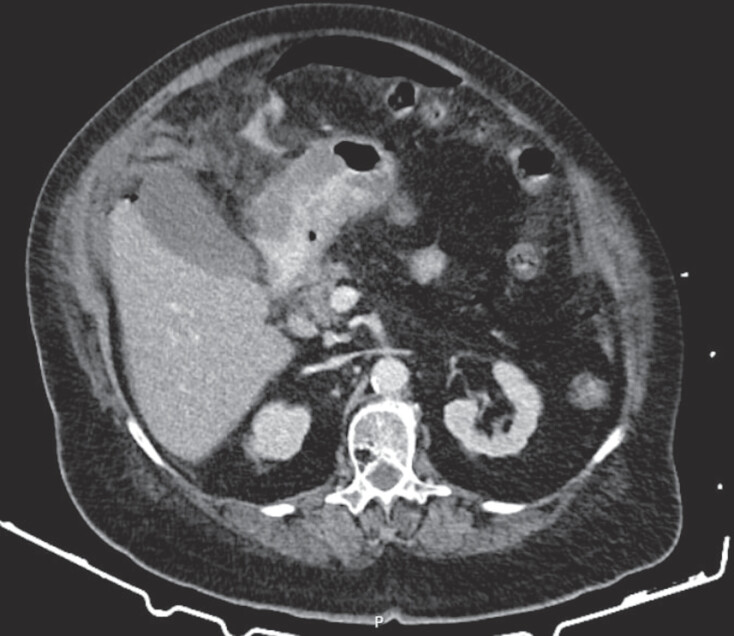
An abdominal computed tomographic scan showing a prepyloric gastric perforation with oral contrast extravasation.

**Fig. 2:**
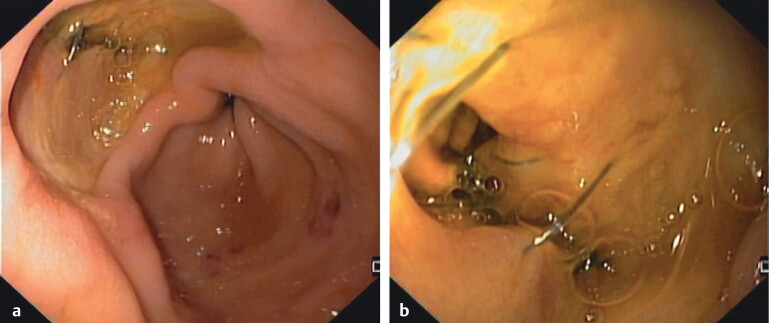
Endoscopic visualization of a large mucosal defect along the anterior antral wall ( **a** ) with disruption of the previous surgical omental patch ( **b** ).

**Fig. 3:**
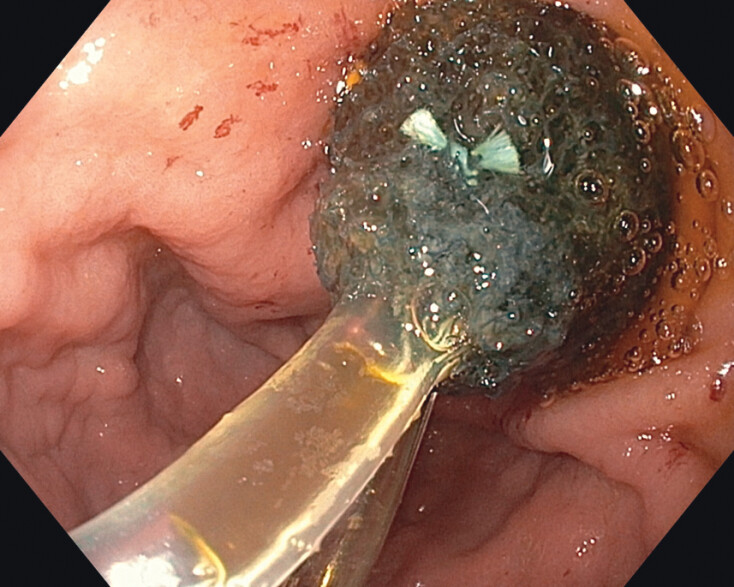
An endoscopic image showing the initial intraluminal placement of the sponge in the antrum.

**Fig. 4:**
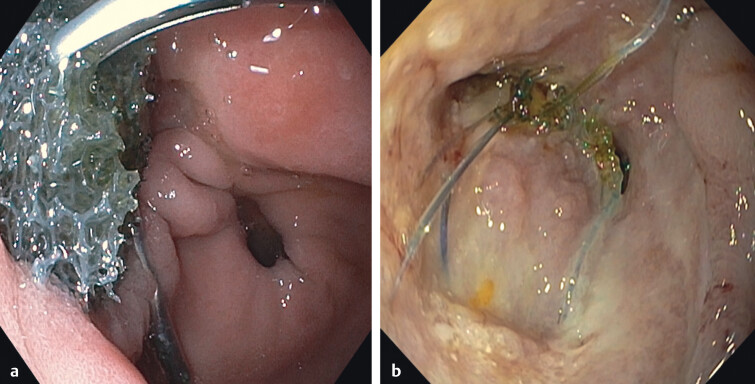
Endoscopic images during endoscopic vacuum therapy, showing the placement of the sponge inside the gastric ulcer ( **a** ) and progressive granulation and reduction of the cavity in the follow-up endoscopy ( **b** ).

**Fig. 5:**
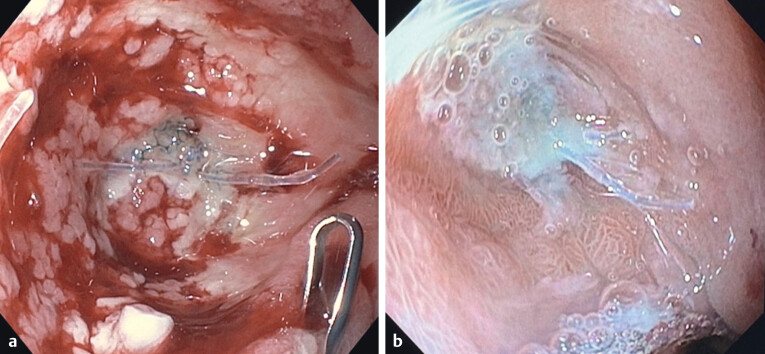
( **a** and **b** ) Final endoscopic appearance showing re-epithelialization and full closure of the defect following 15 days of endoscopic vacuum therapy (two sessions).

A 79-year-old woman with multiple comorbidities presented to the emergency department in shock with acute diffuse abdominal pain and peritoneal irritation. Computed tomography (CT) revealed a perforated prepyloric ulcer ( Fig. 1 ). Patient underwent emergency laparotomy with primary suture of the defect; however, post-procedure was complicated with partial dehiscence of the suture line. Endoscopy revealed a large and excavated ulcer along the anterior antral wall, with a transmural defect in its interior due to the disruption of previous omental patch ( Fig. 2a and b ). Fluoroscopy confirmed contrast leakage. Endoscopic closure of the ulcer was initially attempted with through-the-scope (TTS) clips but failed ( Video 1 ). Given the size of the ulcer and patient’s high surgical risk, endoscopic vacuum therapy (EVT) was attempted. As there was no associated collection for intracavitary treatment, the sponge was initially placed intraluminal at the antrum ( Fig. 3 ); however, proximal migration was seen at follow-up endoscopy, with no granulation effect at the level of the ulcer. Therefore, EVT was repeated, and this time with the sponge located inside the gastric ulcer, after adjusting foam dimensions. TTS clips were placed grabbing the foam and the surrounding mucosa to minimize sponge displacement during endoscopic removal ( Fig. 4a ). Progressive granulation and re-epithelialization were observed during follow-up endoscopy ( Fig. 4b ), with complete epithelialization and leak closure after 15 days of EVT ( Fig. 5a and b ). CT scan confirmed closure of the perforation, with patient resuming oral feeding, being discharged home thereafter.

**Video 1** Endoscopic vacuum therapy is performed to close a transmural gastric defect.


Perforated gastric ulcers have a high mortality risk, particularly among elderly patients with multiple comorbidities. Although surgical repair remains the standard treatment, EVT may be an effective rescue option, particularly in high-risk selected cases. 1​ This case highlights the expanding applications of EVT in the management of perforated gastric ulcers after initial surgical failure.

Endoscopy_UCTN_Code_CPL_1AH_2AG
